# Temperature Dependence of Electrical Resistance in Carbon Nanotube Composite Film during Curing Process

**DOI:** 10.3390/nano12203552

**Published:** 2022-10-11

**Authors:** Fei Xing, Min Li, Shaokai Wang, Yizhuo Gu, Wei Zhang, Yanjie Wang

**Affiliations:** 1 Key Laboratory of Aerospace Advanced Materials and Performance (Ministry of Education), School of Materials Science and Engineering, Beihang University, No. 37 Xueyuan Road, Haidian District, Beijing 100191, China; 2 Research Institute of Frontier Science, Beihang University, No. 37 Xueyuan Road, Haidian District, Beijing 100191, China

**Keywords:** carbon nanotube composite film, process monitoring, temperature dependence, curing shrinkage, thermal expansion

## Abstract

Carbon nanotube (CNT) film possesses excellent mechanical and piezoresistivity, which may act as a sensor for process monitoring and reinforcement of the final composite. This paper prepared CNT/epoxy composite film via the solution dipping method and investigated the electrical resistance variation (Δ*R/R*_0_) of CNT/epoxy composite film during the curing process. The temperature dependence of electrical resistance was found to be closely related to resin rheological properties, thermal expansion, and curing shrinkage. The results show that two opposing effects on electrical resistivity occur at the initial heating stage, including thermal expansion and condensation caused by the wetting tension of the liquid resin. The lower resin content causes more apparent secondary impregnation and electrical resistivity change. When the resin viscosity remains steady during the heating stage, the electrical resistance increases with an increase in temperature due to thermal expansion. Approaching gel time, the electrical resistance drops due to the crosslink shrinkage of epoxy resin. The internal stress caused by curing shrinkage at the high-temperature platform results in an increase in electrical resistance. The temperature coefficient of resistance becomes larger with an increase in resin content. At the isothermal stage, an increase in Δ*R/R*_0_ value becomes less obvious with a decrease in resin content, and Δ*R/R*_0_ even shows a decreasing tendency.

## 1. Introduction

Carbon nanotubes (CNTs) are promising materials to reinforce polymers due to their superior mechanical properties and excellent physical properties. CNTs have been successfully manipulated to form CNT fibers and films with high CNT concentrations. These nanocomposites reinforced by CNT fibers or films even exhibit comparable mechanical properties with traditional carbon fiber reinforced composites [1,2,3], which show great potential as structural materials. For instance, CNT film has been successfully used in the Juno spacecraft by hybridizing it with an M55J carbon fiber [4]. Meanwhile, the prominent electromechanical properties of CNTs and the CNT network also impart the excellent piezoresistive response of CNT composites, which could achieve the strain sensing and structural health monitoring of polymer composites.

The fabrication of thermosetting composites involves complex physical and chemical changes, including resin infiltration, resin curing, fiber densification, and so on. Effective process quality control could avoid the formation of voids, delamination, and other defects. Thereby, process monitoring has attracted more and more attention, and various process monitoring approaches have been developed, such as optical fiber sensors [5], dielectric/capacitance sensors [6], ultrasonic monitoring [7], thermocouples [8], pressure sensors, and resistance sensors. These methods have been successfully used to monitor the heat release of resin curing, curing degree of the matrix, curing shrinkage strain, and gel and glass transition temperature (*T*_g_) of the resin system.

Owing to the remarkable piezoresistivity, CNT materials have also been used for process monitoring, including reinforcement compaction, flow-front tracking, resin gel, and curing [9]. CNT-based sensors may be prepared via different deposition methods, including dip coating, spray coating, in situ growth using chemical vapor deposition (CVD), and electrophoretic deposition (EPD) [2]. Hu et al. fabricated a nanocomposite temperature sensor by dispersing CNTs in a mixture of bisphenol-F epoxy resin and amine hardener, which could monitor temperature change due to the temperature-dependent tunneling effect [10]. Lee et al. also reported an electrical resistance variation caused by a change in the network structure in CNT film [11]. Luo et al. sprayed CNTs onto glass fiber, which was used to sense resin curing in the vacuum-assisted resin transfer molding (VARTM) process according to the electrical resistance variation [12,13,14,15,16]. Gnidakouong et al. realized the monitoring of resin injection, the crosslinking starting point, and the gel point by using MWCNT film during the VARTM manufacturing process [17,18].

Lu et al. implanted CNT buck paper into the middle layer of glass fiber/epoxy prepreg to monitor its electrical resistance change during curing, and the *T*_g_ of the resin was successfully measured [19,20,21]. Most of these CNT sensors need to be placed inside the polymer composite and are embedded inside the final composite after resin curing [22,23]. Those sensors with good piezoresistivity and mechanical properties may achieve structure–function integration. These CNT films synthesized by floating catalyst chemical vapor deposition (FCCVD) have shown outstanding mechanical, electric, and thermal properties with great potential in a broad range of applications [24,25]. FCCVD CNT film has a two-dimensional cross-linked network consisting of numerous overlapped and entangled CNTs [26,27,28]. The CNT film with a high CNT fraction has numerous nanoscale pores, and the solution dipping method has proven to be an effective method to fully infiltrate the CNT film. The random FCCVD CNT film has a tensile strength as high as 598 MPa [29], and its composite film shows a gauge factor of 5.6 under tensile stress [30]. The FCCVD CNT film may be a good candidate to achieve process monitoring during the fabrication process and then act as a reinforcement in the final composites due to their piezoresistivity and excellent mechanical properties.

In this study, the temperature dependence of electrical resistance of FCCVD CNT/epoxy composite film was investigated. To verify the feasibility, validity, and sensitivity of CNT film as a sensor to monitor resin physical and chemical changes, two epoxy systems were used to prepare CNT/epoxy composite films by the solution dipping method, and the influences of resin content and resin type on electrical resistance variation during the curing process were compared. The effects of resin viscosity, thermal expansion, and curing shrinkage on piezoresistivity were further revealed.

## 2. Materials and Methods

### 2.1. Materials

**Figure 1:**
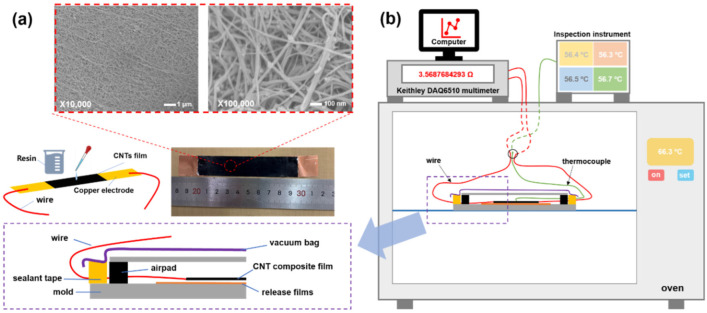
(**a**) The surface morphology of the pristine CNT film, and (**b**) a schematic of in situ monitoring of electrical resistance for CNT/epoxy composite films during curing process.

Carbon nanotube (CNT) film synthesized via FCCVD growth method was used in this research due to its great potential in structure–function integration composites, which was purchased from Suzhou Jiedi Nanotechnology Co., Ltd., Suzhou, Jiangsu Province, China. Figure 1a shows the SEM images of the pristine CNT film, which is a random network formed by entanglement and random orientation of numerous CNTs along the in-plane direction. The CNT film has a thickness of approximately 13 μm, and the average diameter of CNTs is 7.8 nm [3,29]. DGEBA epoxy (E51, CAS#61788-97-4) was chosen as the matrix, which was produced by Nantong Xingchen Synthetic Materials Co., Ltd., Nantong, Jiangsu Province, China. The DGEBA epoxy was cured using curing agents of 1,12-diaminododecane (DAD, CAS#2783-17-7) and ethylenediamine (EDA, CAS#107–15-3), which were purchased from Shanghai Macklin Biochemical Co., Ltd. and Shanghai Aladdin Biochemical Technology Co., Ltd., Shanghai, China, respectively. Conductive silver glue was purchased from Shenzhen Luxianzi Technology Co., Ltd., Shenzhen, Guangdong Province, China. Auxiliary materials for composite film manufacturing, such as vacuum bags, breathers, release films, and peel ply, were purchased from Airtech International Inc., Huntington Beach, CA, USA.

### 2.2. Fabrication of the CNT/Epoxy Composite Film

The pristine CNT film was cut into rectangular strips with dimensions of 20 mm × 100 mm by using a laser marking machine. Epoxy resin E51 was mixed with the curing agent of DAD or EDA at mass ratios of 100:26 and 100:8, respectively. The initial viscosity of the resin was relatively large, and the required amount of resin was very small. Epoxy and acetone solution was prepared at a small resin concentration of 5 wt% to facilitate the resin to penetrate CNT film fully and evenly. According to the mass of CNT strip and resin contents of CNT/epoxy composite film, the resin mass of the corresponding resin solution needed was calculated. CNT/epoxy composite film was prepared via solution dipping method. Different amounts of epoxy solution were applied on CNT strip surface evenly to prepare CNT/epoxy composite film samples with resin weight contents of 30%, 50%, 70%, and 90%. Moreover, the initial resistivities, areal densities, and thicknesses of the pristine CNT film and CNT/epoxy composite films are listed in Table 1. The top and bottom surfaces of the impregnated CNT film were covered with nonporous release film, which was placed in an oven. These samples were all cured at 180 °C for 3 h in an oven, and simultaneously the electrical resistance of CNT film was monitored during curing process.

### 2.3. Characterizations

The temperature dependence of electrical resistance was measured through two wire method by using Keithley DAQ6510 multimeter, as shown in Figure 1b. Copper foil electrodes were bonded at the two sides of CNT film strip by using conductive silver glue, and the gauge length was controlled at 10 mm. Two thermocouples were attached to CNT/epoxy composite film surface to monitor the real-time temperature. The electrical resistance variation (Δ*R/R*_0_) was characterized every second during the whole curing process, where Δ*R* was the difference between the electrical resistance at a certain time and initial electrical resistance (*R*_0_). The microstructure of CNT/epoxy composite film was observed by using Apreo S LoVac thermal field emission scanning electron microscope.

The curing characteristic of resin system was measured by using a synchronous thermal analyzer (Mettler Toledo), which was heated from room temperature to 200 °C. The rheological property of the resin system was tested by using Gemini200 rotary rheometer (Bohlin Instrument). The parallel plate oscillation mode was used at an oscillation frequency of 1 Hz and controlled stress of 1 Pa. The resin system was heated from room temperature to resin gel temperature at 5 °C /min. The specific surface areas and mesoporous diameters of the pristine CNT film and the CNT composite films were measured by using Quantachrome NovaWin instruments, with BET and BJH methods in nitrogen. The relative volume change (Δ*v/v*_0_) of resin system during curing process was measured by using glass measuring cylinders. Both silicone oil and resin system were poured into measuring cylinders successively. The volume change was recorded by reading the scale of silicon oil surface.

## 3. Results

### 3.1. Temperature Dependence of Electrical Resistance for CNT/E51-DAD Composite Film during Curing Process

**Figure 2:**
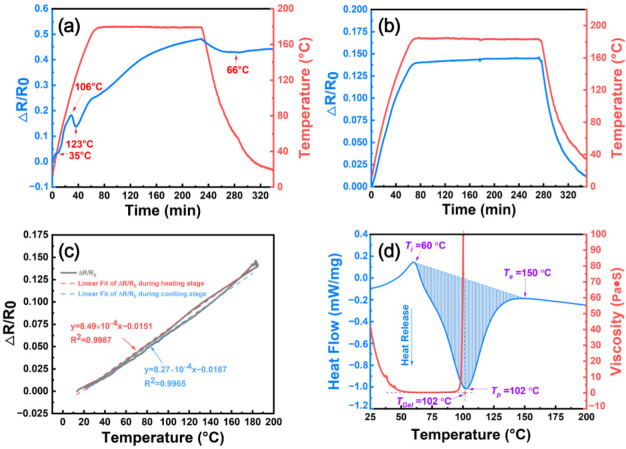
(**a**)The variation in electrical resistance and temperature with time during curing process for CNT/E51-DAD-90 sample, the electrical resistance variation with (**b**) time and (**c**) temperature for pristine CNT film, and (**d**) the viscosity and heat flow with temperature for E51-DAD resin system.

Figure 2a shows the electrical resistance variation (Δ*R/R*_0_) of the CNT/epoxy composite film and the temperature change with time during the curing process. This CNT/epoxy composite film is impregnated with E51-DAD resin, which has a resin content of 90 wt%. The impregnated CNT film is firstly heated up to 180 °C with a heating rate of 6 °C/min, and then the temperature is held at 180 °C for 3 h. Subsequently, the composite film is cooled down at 1.5 °C/min. The initial resistance (*R*_0_) of the CNT/E51-DAD-90 sample with a dimension of 100 mm × 20 mm is measured to be 4.55 Ω at ambient temperature. According to Figure 2a, some inflection points are observed at around 35 °C, 50 °C, 106 °C, and 123 °C respectively, where the Δ*R/R*_0_ profile shows various slopes at the heating stage. Its Δ*R/R*_0_ firstly increases monotonously to 18.21% with increasing temperature until 106 °C. From 106 °C to 123 °C, Δ*R/R*_0_ shows a decreasing tendency to 13.73% at 123 °C. After that, the Δ*R/R*_0_ of the composite film continues to increase considerably with increasing temperature. At the isothermal stage of 180 °C, the Δ*R/R*_0_ keeps increasing up to 48.09%, corresponding to the peak value during the whole process. At the cooling stage, the Δ*R/R*_0_ starts to decrease slightly to 42.93% till 66 °C and then shows a slight increase at the final stage to room temperature. These complex change tendencies of the Δ*R/R*_0_ curve of CNT/E51-DAD-90 should be ascribed to the infiltration, thermal physical, and thermal chemical reactions of the epoxy resin, which exert a determinate impact on the electrical conductivity of the CNT/epoxy composite film.

Different from the CNT/epoxy composite film, the Δ*R/R*_0_ of the pristine CNT film exhibits a strong linear relationship with temperature during both the heating and cooling stages, as displayed in Figure 2b,c. A positive temperature coefficient (PTC) is estimated to be 8.49 × 10^−4^ °C^−1^ for the CNT film, which relates to the distinct resistance changes in the CNT network and the inside iron catalyst [3]. At 180 °C, the Δ*R/R*_0_ of the pristine CNT film has a maximum value of 14.20%, which is only 30 percent of the maximum Δ*R/R*_0_ value, namely 48.09%, of CNT/E51-DAD-90. It is well known that the piezoresistive effect of the CNT network originates from the variation in the intrinsic resistance of CNT (*R*_i_), the direct contact resistance between CNTs (*R*_c_), and the tunnel resistance (*R*_t_) [2]. Following the specific fabrication process of CNT/E51-DAD-90, the infiltration of epoxy resin into the CNT network, the gelation of the resin, the thermal expansion, and the curing shrinkage could all cause the electrical resistance change in the composite film. This indicates that the CNT film has a potential application for monitoring the composite internal micron structure behavior during the processing process.

In order to reveal the inside mechanisms of electrical resistance variation during curing, the rheological properties, curing shrinkage, and curing behavior of the E51-DAD resin system were measured, as shown in Figure 2d. From room temperature to about 50 °C at the heating stage, resin mainly occurs during thermal expansion and viscosity change. On the one hand, the increasing temperature results in the volume expansion of epoxy resin, which increases the *R*_c_ between CNTs. On the other hand, the rheological curve shows that the viscosity of E51-DAD resin decreases from 60.0 Pa·s at ambient temperature to a relatively stable low value of 1.0 Pa·s after 50 °C. The lower viscosity at elevated temperature is beneficial to the resin impregnation into nanoscale pores in the CNT film, and, thus, the impregnated CNT film is further condensed due to the wetting tension of the liquid resin, which results in a decrease in electrical resistance. These two opposing effects jointly cause a mild increase in electrical resistance when the temperature rises from 35 °C to 50 °C. When the temperature continues increasing, the E51-DAD resin begins to gel, and the largest change in the curing degree is caused at 102 °C, and correspondingly the electrical resistance drops slightly in the range of 106–123 °C.

**Figure 3:**
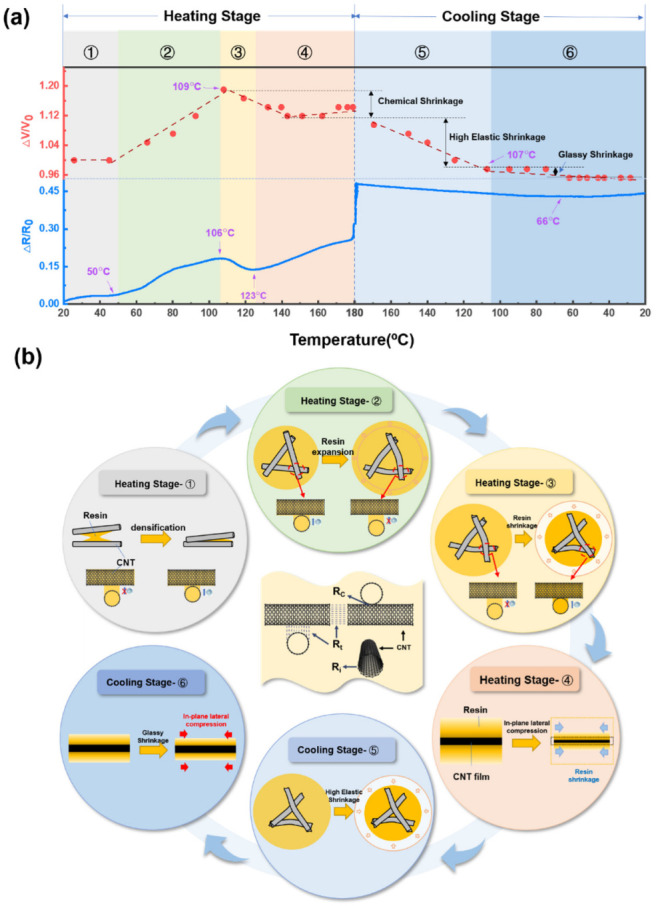
(**a**) The variation in electrical resistance of CNT/E51-DAD-90 and the relative volume change with temperature for E51-DAD resin system, and (**b**) illustrations of the electrical resistance evolution of CNT/epoxy composite film during curing process.

Figure 3a shows the relative volume change and the variation in electrical resistance with temperature for the E51-DAD resin system. The illustrations in Figure 3b present the resistance evolution mechanism of the CNT/epoxy composite film during different stages of the curing process. The epoxy resin experienced a viscous flow state, rubbery state, and glassy state during the curing process. The curing reaction is irreversible, and the residual stress in the curing process was maintained in the CNT composite film. In addition, the coefficients of thermal expansion are different in the rubbery state and the glassy state. These physical and chemical changes cause different electrical resistances at the heating and cooling stages, even at the same temperature. For the CNT/epoxy composite film, the CNT network is partially or fully infiltrated by resin. The thermal expansion of the polymer matrix might increase the distance between CNTs, causing an increase in *R*_c_, as shown in Figure 3b. After 50 °C, the relative volume change of the E51-DAD resin during the curing process indicates that thermal expansion plays a more important role in electrical resistance change, and the slope of Δ*R/R*_0_ and the temperature plot becomes higher than that at low temperature. Although the curing reaction occurs when the temperature reaches 60 °C, as shown in the DSC curve, the resin volume increases by 19.0% in the range of 50–106 °C due to thermal expansion, which explains the increase in electrical resistance before 106 °C. Meanwhile, at an elevated temperature range of 106 °C to 180 °C, the volume of the resin system decreases by 7.1% due to curing shrinkage. The curing shrinkage shortens the CNT-to-CNT distance, which is beneficial to electrical conduction. Afterward, internal stress caused by curing shrinkage is accumulated inside the thin CNT/epoxy composite film, which induces in-plane lateral compression. The electrical resistance increases with an increase in the absolute value of compressive strain [31]. As a result, Δ*R/R*_0_ value increases to 12.03% from 123 °C to 180 °C. This phenomenon also suggests that the CNT film has the potential to detect residual stress as a sensor in polymer composites.

At the isothermal stage, the value of Δ*R/R*_0_ increases up to 48.09%. A similar phenomenon was observed when reduced graphene oxide (RGO) was sprayed onto carbon fiber [14]. This could be caused by the delayed response of shrinkage stress concentration. The Δ*R/R*_0_ amplification at the first 85 min and the following 95 min of the isothermal stage is 15.21% and 7.57%, respectively. This indicates that the stress concentration effect gradually decreases, which is influenced by the resin type and resin content. During the cooling stage, the volume of polymer resin drops by 14.3% from 180 °C to 107 °C and then slowly drops by 2.4% from 107 °C to room temperature, while the turning point corresponds to *T*_g_. The matrix shrinkage may cause a decrease in electrical resistance. In the glassy state, due to smaller thermal expansion than that in the high elastic state, the accumulated internal stress may result in a slight increase in resistance. From 66 °C to room temperature, the Δ*R*/*R*_0_ of the CNT/E51-DAD-90 shows a slight increase because the generation of internal stress causes in-plane lateral compression.

**Figure 4:**
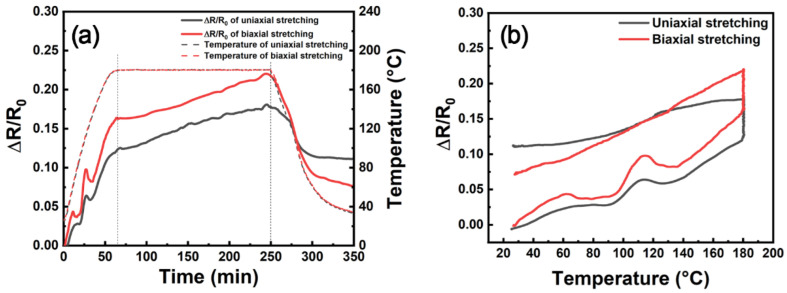
The electrical resistance variation with (**a**) time and (**b**) temperature for stretched samples.

**Figure 5:**
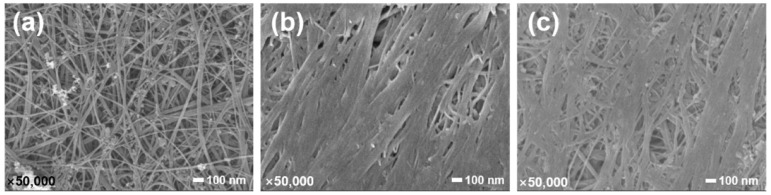
The surface morphology of the pristine CNT film (**a**) and uniaxial (**b**) and biaxial (**c**) stretching samples.

In order to investigate the effect of CNT dispersion on piezoresistive performance, we changed the CNT network structure through uniaxial and biaxial stretching methods [29]. The CNT film was stretched by using a universal mechanical testing machine. For the uniaxial stretching sample, the original specimen had a dimension of 10 cm × 5 cm, which was stretched at a speed of 0.5 mm/min. For the biaxial stretching sample, a square sample was prepared with a dimension of 10 cm × 10 cm. The sample was stretched on the *x*-axis and *y*-axis with stretching ratios of 3.5%. E51-DAD was used to infiltrate the stretched film with a resin mass fraction of 90%. Compared with the pristine CNT film, the CNTs in the uniaxially stretched sample are partially oriented along the stretching direction, and the pores of the biaxially stretched sample become larger. The electrical resistance variations with time and temperature for these stretched samples are shown in Figure 4. Compared with the CNT/E51-DAD-90 film in Figure 2a and Figure 3, the resistance variation curves show similar profiles, but the Δ*R/R*_0_ value of the stretched sample is smaller than that of the pristine CNT film. From the SEM morphology in Figure 5, it can be seen that CNTs formed bundles after stretching, and the bundle structure is averse to piezoresistive performance.

### 3.2. Effect of Resin Content on the Temperature Dependence of Electrical Resistance of CNT/E51-DAD Composite Film

**Figure 6:**
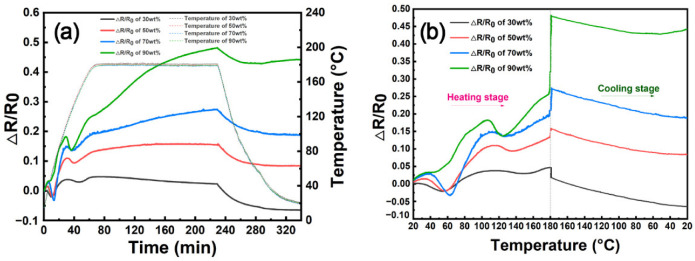
The electrical resistance variation with (**a**) time and (**b**) temperature for CNT/E51-DAD samples with different resin content during curing process.

In order to further investigate the effect of resin content on the temperature dependence of electrical resistance of CNT/epoxy composite film, CNT/E51-DAD samples with resin contents of 30 wt%, 50 wt%, 70 wt%, and 90 wt% were prepared, which were denoted as CNT/E51-DAD-30, CNT/E51-DAD-50, CNT/E51-DAD-70, and CNT/E51-DAD-90, respectively. Figure 6a,b shows the resultant Δ*R/R*_0_ variation with time and temperature. It can be seen that the Δ*R/R*_0_ value drops at 37 ± 2 °C for CNT/E51-DAD-30, CNT/E51-DAD-50, and CNT/E51-DAD-70 films, and the minimum values are even smaller than *R*_0_. This could be related to the secondary infiltration caused by the wetting tension of the liquid resin of resin in CNT network.

**Figure 7:**
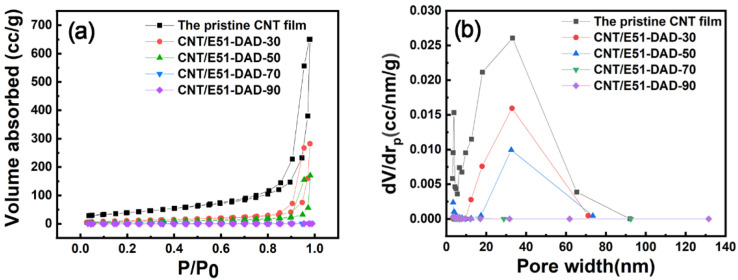
(**a**) N_2_ adsorption–desorption isotherm curves and (**b**) pore size distribution curves of the pristine CNT film and CNT composite films after infiltrating the E51-DAD resin.

**Figure 8:**
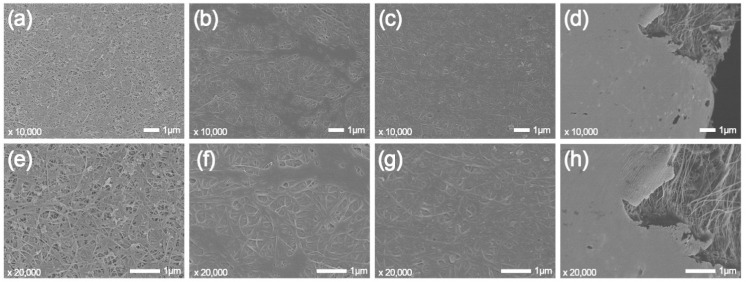
The surface morphology of the CNT/E51-DAD films with resin contents of (**a**) 30 wt%, (**b**) 50 wt%, (**c**) 70 wt%, (**d**) 90 wt% at 10,000 times and (**e**) 30 wt%, (**f**) 50 wt%, (**g**) 70 wt%, (**h**) 90 wt% at 20,000 times.

On the one hand, the viscosity of resin becomes smaller with increasing temperature, and resin is easier to be squeezed out from CNT bundles to these nanopores under vacuum pressure. Consequently, the CNT film is densified, and the electrical resistance decreases. The N_2_ adsorption–desorption isotherm curves and pore size distribution curves of the CNT/E51-DAD composite films are shown in Figure 7. As exhibited in Table 2, the pristine CNT film has a specific surface area as high as 142.5 m^2^/g. The porosity diameter of the CNT/E51-DAD-30 sample remains at a large value of 32.9 nm, and the CNT network is partially infiltrated. As shown in Figure 8, with an increase in resin content, the coated resin becomes thicker, and the pore becomes smaller. For CNT/E51-DAD-30, CNT/E51-DAD-50, and CNT/E51-DAD-70, small pores and CNT texture can be observed from the surface morphology, while the CNT network has been fully filled in CNT/E51-DAD-90. After resin impregnation, the resin is preferably coated on CNTs. Although the CNT/epoxy composite film still shows a porous structure, the specific surface area of the CNT/E51-DAD composite film decreases greatly after resin infiltration, and the porosity of the network structure is reduced. On the other hand, the coefficient of thermal expansion (CTE) of the resin system is much higher than the CNT film [3], which may cause an increase in *R*_c_ during the heating process. The enhanced fluidity of polymer resin at an elevated temperature promotes its imbibition inside the CNT film. The fully impregnated CNT/E51-DAD-90 film has a small porosity diameter of 3.8 nm and a low specific surface area of 0.1 m^2^/g. A higher resin content results in a faster reaction rate, and a low resin content has a certain hysteresis phenomenon to the characteristic temperature. The thermal expansion effect plays a more important role in electrical resistance change than the densification effect. Therefore, the Δ*R/R*_0_ value of the CNT/E51-DAD-90 sample does not drop at this stage.

When the temperature continues to increase from around 50 °C to 102 °C, the electrical resistance shows an increasing tendency due to thermal expansion, as expected. The Δ*R/R*_0_ values of CNT/E51-DAD-30, CNT/E51-DAD-50, CNT/E51-DAD-70, and CNT/E51-DAD-90 reach 3.79%, 10.93%, 14.75%, and 18.21% when the temperature rises to around 102 °C. The effect of thermal expansion on the temperature dependence of electrical resistance becomes more significant with an increase in resin content. After the resin gel point at around 102 °C, Δ*R/R*_0_ decreases with increasing temperature, and Δ*R/R*_0_ reductions are 0.83%, 1.55%, 1.15%, and 4.48% for CNT/E51-DAD-30, CNT/E51-DAD-50, CNT/E51-DAD-70, and CNT/E51-DAD-90, respectively. A higher resin content results in greater curing shrinkage and more obvious electrical resistance change. After the rapid curing stage around *T*_p_, Δ*R/R*_0_ increases with an increase in temperature until the isothermal stage at 180 °C. The temperature coefficient of resistance (TCR) is usually used to describe the temperature dependence of electrical resistance, which is equal to the variation in electrical resistance when the temperature is changed by 1 K [10,32]. The TCRs of CNT/E51-DAD-30, CNT/E51-DAD-50, CNT/E51-DAD-70, and CNT/E51-DAD-90 are calculated to be 2.89 × 10^−4^, 8.44 × 10^−4^, 12.1 × 10^−4^, and 16.1 × 10^−4^ °C^−1^. The TCRs of these CNT composites in this paper were compared with the results in the reported literature, as shown in Table 3. It should be pointed out that the TCRs of the CNT composite films in this paper were lower than MWCNT-coated fabrics or MWCNT buck paper. The shorter, looser accumulation and lower content of CNT aggregations usually have higher TCR values [3,10,32,33,34]. However, CNT film is an ideal reinforcement for structure–function integration applications, which has a tensile strength and modulus of 186 MPa and 3.2 GPa [29], respectively. CNT/E51-DAD films show similar TCRs to that of 3.6 × 10^−4^ °C^−1^ of MWCNT/epoxy composite film synthesized by CCVD, which also had a high CNT purity of >95% [33,34], which suggests that the CNT film has excellent temperature dependence of electrical resistance to monitor the composite forming process and has the potential for the structure–function integration of composites.

At the isothermal stage, an interesting phenomenon is found, that is, Δ*R/R*_0_ values show a contrary tendency for those CNT films with different resin contents. The Δ*R/R*_0_ value of the CNT/E51-DAD-30 sample decreases by 2.29% after 3 h at 180 °C, while the Δ*R/R*_0_ values of CNT/E51-DAD-50, CNT/E51-DAD-70, and CNT/E51-DAD-90 increase 1.97%, 8.09%, and 22.33%. The DSC curve of Figure 2d shows that E51-DAD resin is almost fully cured, and so the resin curing shrinkage is almost at the end at the isothermal stage. However, due to the delayed response caused by polymer viscoelasticity, the stress concentration effect works at the isothermal stage [14]. The continuous increase or decrease in the Δ*R/R*_0_ is mainly dependent on the stress concentration effect.

During the cooling stage, the electrical resistance of the CNT/E51-DAD film decreases with decreasing temperature, and Δ*R/R*_0_ decreases more slowly when the temperature is higher than the glass transition temperature of 107 °C. The Δ*R/R*_0_ reduction values of different CNT/E51-DAD films are close.

### 3.3. Effect of Matrix Property on the Temperature Dependence of Electrical Resistance of CNT/Epoxy Composite Film

**Figure 9:**
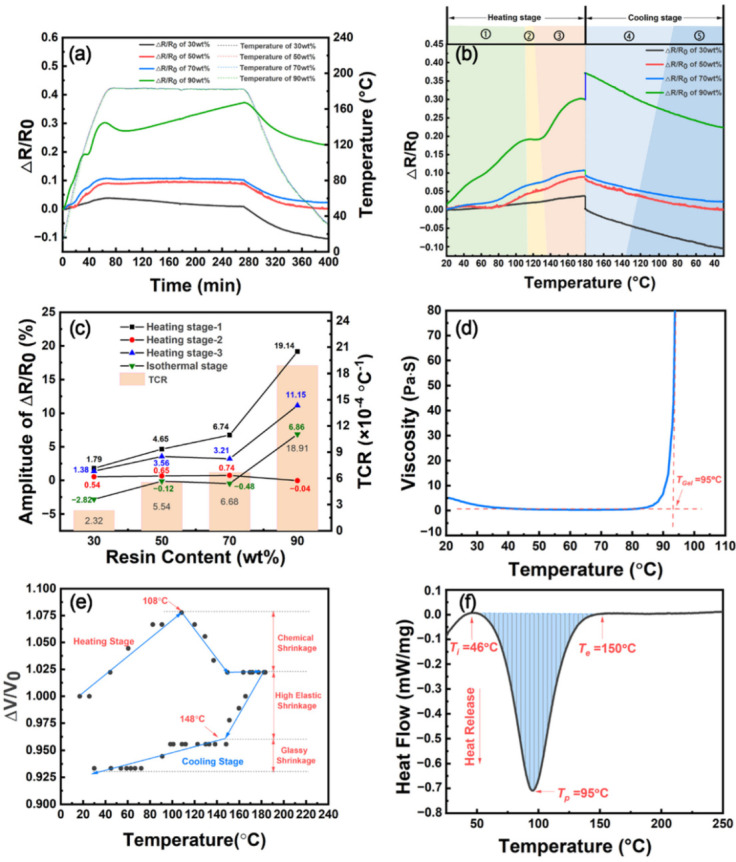
The electrical resistance variation with (**a**) time and (**b**) temperature for CNT/E51-EDA samples with different resin contents during curing process; (**c**) the amplitude of the electrical resistance variation and the TCR for CNT/E51-EDA samples with different resin contents; and (**d**) the resin viscosity, (**e**) the relative volume change, and (**f**) the heat flow with temperature for E51-EDA.

Figure 9a,b shows the Δ*R/R*_0_ change with time and temperature for CNT/E51-EDA composite film during the curing process. Both EDA and DAD are binary amine curing agents. Owing to the shorter fatty chain of EDA, the crosslinking density and the shrinkage ratio of E51-EDA are higher than E51-DAD. The internal stress of E51-EDA is higher than E51-DAD resin. For comparison, CNT/E51-EDA samples with resin contents of 30 wt%, 50 wt%, 70 wt%, and 90 wt% were prepared. Figure 9c shows the amplitude of the electrical resistance variation and the TCR for CNT/E51-EDA samples with different resin contents. The Δ*R/R*_0_ curves of the CNT/ E51-EDA samples show a similar profile as CNT/E51-DAD, except the Δ*R/R*_0_ drop in the temperature range of 35–60 °C.

Figure 9d shows the viscosity-temperature curve of the E51-EDA resin system. CNT/E51-EDA and CNT/E51-DAD have viscosities of 5.1 and 60.0 Pa·s at ambient temperature, respectively. The low viscosity of E51-EDA is beneficial to resin impregnation into the CNT film at ambient temperature, and, thereby, resin fluidity at elevated temperature has less influence on CNT/E51-EDA. As a result, the electrical resistance changes in CNT/E51-EDA are mainly dominated by thermal expansion of the resin system. At around 100 °C, turning points are observed for the CNT/E51-EDA sample due to curing shrinkage. Among these samples, CNT/ E51-EDA-90 shows the biggest Δ*R/R*_0_ drop of 0.04%, which is smaller than the CNT/ E51-DAD-90 sample. This is closely related to the curing shrinkage ratio of E51-DAD and E51-EDA, which are 7.5% and 5%, respectively, as shown in Figure 3 and Figure 9e. The DSC curves in Figure 2d and Figure 9f show that the curing heat release of E51-DAD and E51-EDA resin are 469.81 and 296.66 mJ/mg, respectively. Thermal expansion plays a more important role than curing shrinkage for E51-EDA.

At the isothermal stage, the electrical resistances of the CNT/epoxy composite films show decreasing tendency except CNT/E51-EDA-90 as CNT/E51-DAD-50, CNT/E51-DAD-70, and CNT/E51-DAD-90. Among these samples, CNT/E51-EDA-30 shows the biggest Δ*R/R*_0_ drop of 2.82% at 180 °C for 3 h. The Δ*R/R*_0_ value of CNT/E51-DAD-90 increases by 6.86% at 180 °C for 3 h. This also indicates that the resin system with less shrinkage ratio yields smaller residual stress and Δ*R/R*_0_ change. During the cooling stage, the Δ*R/R*_0_ of the CNT/E51-EDA also shows decreasing tendency as CNT/E51-DAD. The *T*_g_ of the E51-EDA is measured to be 152 °C, and no obvious slope change is observed for the sample with higher *T*_g_. Therefore, the monitoring effect of CNT film is similar for different resin systems. During the process of composite molding, the curing process of the CNT film and resin system of the composite is premonitored in advance, which can effectively guide the molding quality of composite materials in the actual molding process.

## 4. Conclusions

This paper mainly focuses on the electrical resistance variation in FCCVD CNT film, which can act as a sensor for process monitoring and reinforcement in the final composites due to its excellent mechanical property and temperature dependence of electrical resistance. The CNT/E51-DAD and CNT/E51-EDA were prepared via the solution dipping method, and the temperature dependence of electrical resistance of these CNT composite films was investigated to reveal the influences of resin content and resin type on electrical resistance variation during the curing process. The inside mechanisms were further revealed by investigating the characteristics of resin viscosity, thermal expansion, and curing shrinkage during the curing process. It was found that two opposing effects on electrical resistance occur at the initial heating stage, including the thermal expansion and condensation caused by secondary impregnation. The lower resin content causes more obvious secondary impregnation and electrical resistivity change. When the resin viscosity is stabilized after 50 °C, the electrical resistance increases with an increase in temperature due to thermal expansion. Around the gel point, the electrical resistance drops due to curing shrinkage. The CNT/E51-DAD-90 sample shows the biggest Δ*R/R*_0_ drop than CNT/E51-EDA-90 in this stage, which is caused by the high shrinkage ratio of E51-DAD. In addition, the CNT bundle structure is averse to piezoresistive performance. The internal stress caused by curing shrinkage at a high-temperature platform induces in-plane lateral compression, resulting in an increase in electrical resistance. The temperature coefficient of resistance becomes bigger with an increase in resin content. At the isothermal stage, the increase in Δ*R/R*_0_ value becomes less obvious with a decrease in resin content, and Δ*R/R*_0_ even shows a decreasing tendency in the CNT/E51-DAD-30 sample. The E51-EDA samples show the same electrical response, which is slightly different due to the different viscosity, exothermic heat, glass transition, and so on.

## Figures and Tables

**Table 1:** The initial resistivities, the areal densities, and the thicknesses of the pristine CNT film and CNT/epoxy composite films before curing.

Sample	Initial Resistivity/×10^−5^ Ω·m	Areal Density/×10^−4^ g·cm^−2^	Thickness/μm
The pristine CNT film	1.152 ± 0.005	8.6 ± 0.6	13 ± 2
CNT/E51-DAD-30	1.445 ± 0.006	8.7 ± 0.4	10 ± 2
CNT/E51-DAD-50	1.576 ± 0.003	14.2 ± 0.5	14 ± 1
CNT/E51-DAD-70	1.614 ± 0.004	25.0 ± 0.8	17 ± 3
CNT/E51-DAD-90	2.740 ± 0.008	72.0 ± 1.1	30 ± 4
CNT/E51-EDA-30	1.709 ± 0.001	10.6 ± 0.3	13 ± 2
CNT/E51-EDA-50	2.752 ± 0.001	18.0 ± 0.2	21 ± 3
CNT/E51-EDA-70	3.785 ± 0.003	34.0 ± 0.6	30 ± 3
CNT/E51-EDA-90	6.957 ± 0.003	91.1 ± 1.2	35 ± 4

**Table 2:** The specific surface areas and pore diameters of the pristine CNT film and the CNT composite films after infiltrating the E51-DAD resin.

Sample	S_BET_/m^2^·g^−^^1^	S_BJH_/m^2^·g^−1^	V_BJH_/cc·g^−1^	Pore Diameter/nm
The pristine CNT film	142.5	168.6	1.0	33.2
CNT/E51-DAD-30	42.5	60.9	0.5	32.9
CNT/E51-DAD-50	25.9	33.5	0.3	32.6
CNT/E51-DAD-70	0.5	0.5	0.002	3.8
CNT/E51-DAD-90	0.1	0.7	0.001	3.8

**Table 3:** Comparison of TCRs of different CNT composite materials [10,19,20,21,32,33,34].

CNT Aggregations	Temperature Range/°C	Process Method	CNT Content	TCR/×10^−4^ °C^−1^	Ref.
MWCNT/epoxy	60–100	Mixing dispersion	5 wt%	210	[10]
MWCNTs buck paper	20–127	Centrifugation and filtration	--	−5.6	[19]
MWCNTs buck paper	23–108	Centrifugation and filtration	--	About 267	[20]
GNP */MWCNT film	RT **-140	Stir-sonicated spray vacuum	MWCNT: GNP = 10:1	About 86.7	[21]
MWCNT	30–130	CVD deposition	100	−1.9~6.4	[32]
SWCNT/PI ***	RT-500	Dielectrophoresis deposition	--	−150~100	[33]
MWCNT/epoxy	RT-160	Mixing dispersion	0.5 wt%	3.6	[34]
FCCVD CNT film/epoxy	RT-180	Solution dipping	70, 50, 30, 10 wt%	2.32–18.91	Our work

* GNP, graphene nanoplate; ** RT, room temperature; *** PI, polyimide.

## Data Availability

The data presented in this study are available on request from the corresponding author.
